# Influence of Strain Gradient on Fatigue Life of Carbon Steel for Pressure Vessels in Low-Cycle and High-Cycle Fatigue Regimes

**DOI:** 10.3390/ma15020445

**Published:** 2022-01-07

**Authors:** Tomoyuki Fujii, Muhamad Safwan Bin Muhamad Azmi, Keiichiro Tohgo, Yoshinobu Shimamura

**Affiliations:** 1 Department of Mechanical Engineering, Shizuoka University, Hamamatsu 432-8561, Japan; tohgo.keiichiro@shizuoka.ac.jp (K.T.); shimamura.yoshinobu@shizuoka.ac.jp (Y.S.); 2 Faculty of Mechanical Engineering Technology, Pauh Putra Campus, Universiti Malaysia Perlis (UniMAP), Arau 02600, Malaysia; safwanazmi@unimap.edu.my

**Keywords:** fatigue testing, bending, local strain concepts, elastically/plastically dominant fatigue

## Abstract

This paper discusses how the strain gradient influences the fatigue life of carbon steel in the low-cycle and high-cycle fatigue regimes. To obtain fatigue data under different strain distributions, cyclic alternating bending tests using specimens with different thicknesses and cyclic tension–compression tests were conducted on carbon steel for pressure vessels (SPV235). The crack initiation life and total failure life were evaluated via the strain-based approach. The experimental results showed that the crack initiation life became short with decreasing strain gradient from 10^2^ to 10^6^ cycles in fatigue life. On the other hand, the influence of the strain gradient on the total failure life was different from that on the crack initiation life: although the total failure life of the specimen subjected to cyclic tension–compression was also the shortest, the strain gradient did not affect the total failure life of the specimen subjected to cyclic bending from 10^2^ to 10^6^ cycles in fatigue life. This was because the crack propagation life became longer in a thicker specimen. Hence, these experimental results implied that the fatigue crack initiation life could be characterized by not only strain but also the strain gradient in the low-cycle and high-cycle fatigue regimes.

## 1. Introduction

Fatigue failures in engineering components are a serious problem in machines and structures. To increase the structural integrity of components, many studies have investigated fatigue mechanisms, fatigue life, and life prediction techniques for structural materials. The fatigue life of metallic materials is classified into two types depending on the number of cycles to failure: a fatigue life below roughly 10^4^ cycles is called low-cycle fatigue (LCF), and a longer fatigue life is called high-cycle fatigue (HCF). The Great East Japan Earthquake in 2011 triggered much research on LCF to assess the structural integrity of power plants against earthquake motions.

**Figure 1:**
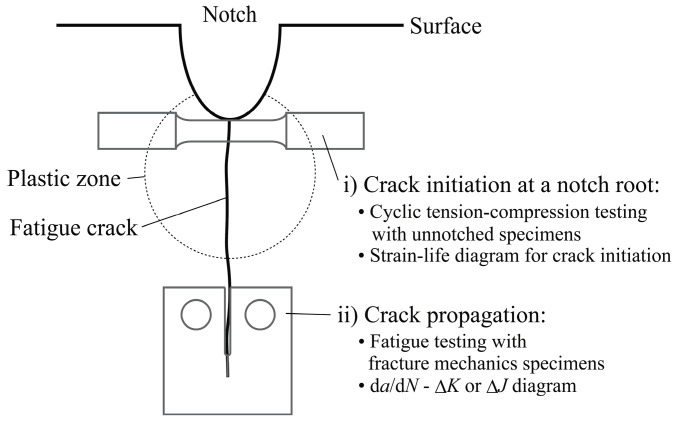
Schematic illustration of fatigue life evaluation for a notched component.

The LCF life of components is generally evaluated on the basis of a combination of the durations of crack initiation and crack propagation [1]. Figure 1 shows a schematic illustration of a life evaluation technique for a notched component. For crack initiation, stress–strain hysteresis at a notch root that is plastically deformed due to an applied load is analyzed, and the crack initiation life is estimated based on the LCF data of a material obtained on smooth (unnotched) specimens under cyclic uniaxial loading, such as tension–compression (T/C). For crack propagation, a fracture mechanics parameter, such as stress intensity factor *K* or *J*-integral for a crack propagating from the notch root, is analyzed, and the crack propagation life is estimated based on the crack propagation properties of the material (d*a*/d*N*-Δ*K* or d*a*/d*N*-Δ*J* relations). If the evaluation of crack initiation life is made using the above procedure, stress–strain distributions near a stress/strain raiser are ignored. Although a few studies related to LCF life evaluation considering a strain gradient have been recently reported [2,3], the understanding of the influence of strain distribution on fatigue life is still insufficient to apply it to the life prediction of components in the LCF regime. In contrast, it is well known that stress distribution affects fatigue life in the HCF regime, and Peterson [4], Neuber [5], Ishibashi [6], and Siebel et al. [7] formulated the fatigue limit taking the stress gradient into account. Recently, Ye et al. [8] proposed a technique to predict the fatigue life of notched specimens taking into account stress and its gradient. They demonstrated that the fatigue life of notched specimens with different notch sizes could be evaluated using this technique.

In a smooth specimen, the specimen thickness affects the fatigue life, which is a so-called size effect, due to the stress gradient at the specimen surface. A few studies on bending fatigue testing in the LCF regime have been conducted to evaluate the size effect and the influence of strain gradient on fatigue failure life. Kulesa et al. [9], Kurek et al. [10], and Kurek [11] developed a lab-made bending testing apparatus to conduct strain-controlled cyclic bending and evaluated the relationship between fatigue failure life and total strain amplitude. They demonstrated that the fatigue life of bending specimens was slightly longer than that of T/C specimens. Bending fatigue testing not only enables the investigation of the influence of the strain gradient on fatigue life but also has the advantage that the bending fatigue testing can be performed at a lower load than the T/C fatigue testing. It should be noted that there is a problem with cyclic bending in the LCF regime: if a specimen is plastically deformed, it is difficult experimentally to measure mechanical quantities of stress, elastic strain, and plastic strain, although the total strain can be easily measured [12]. That is, the effectiveness of the life assessment based on the strain-based approach of the Basquin law [13] and the Coffin–Manson law [14,15] for mechanical components subjected to cyclic bending remains to be clarified. Karolczuk et al. [16,17] developed a numerical technique for calculating the mechanical quantities in a plastically deformed specimen subjected to bending on the assumption that the bending strain distribution along the specimen thickness was linear. Fujii et al. [18] developed a technique to obtain these quantities by a combination of experiment and elastic–plastic finite element analysis (FEA). They conducted alternating four-point bending testing and evaluated fatigue life in the LCF and HCF regimes using a strain-based approach. Then, the obtained fatigue data were compared with the life estimated by the universal slope method [19], which is based on tensile testing data. This comparison implied that the strain gradient would affect fatigue life, irrespective of load cycles. To clarify the validity of this result related to the influence of the strain gradient on fatigue life in an LCF regime, it is necessary to compare the results obtained by the experiments.

The present study aims to clarify the influence of the strain gradient on the fatigue life of a carbon steel. Cyclic alternating bending tests using specimens with different thicknesses were conducted, and cyclic T/C tests were also performed. The crack initiation life and total failure life were evaluated using the strain-based approach of the Basquin law and the Coffin–Manson law. Then, the relationship between strain gradient and fatigue life is discussed based on the experimental results obtained from the different fatigue tests.

## 2. Materials and Methods

### 2.1. Specimen Material

**Figure 2:**
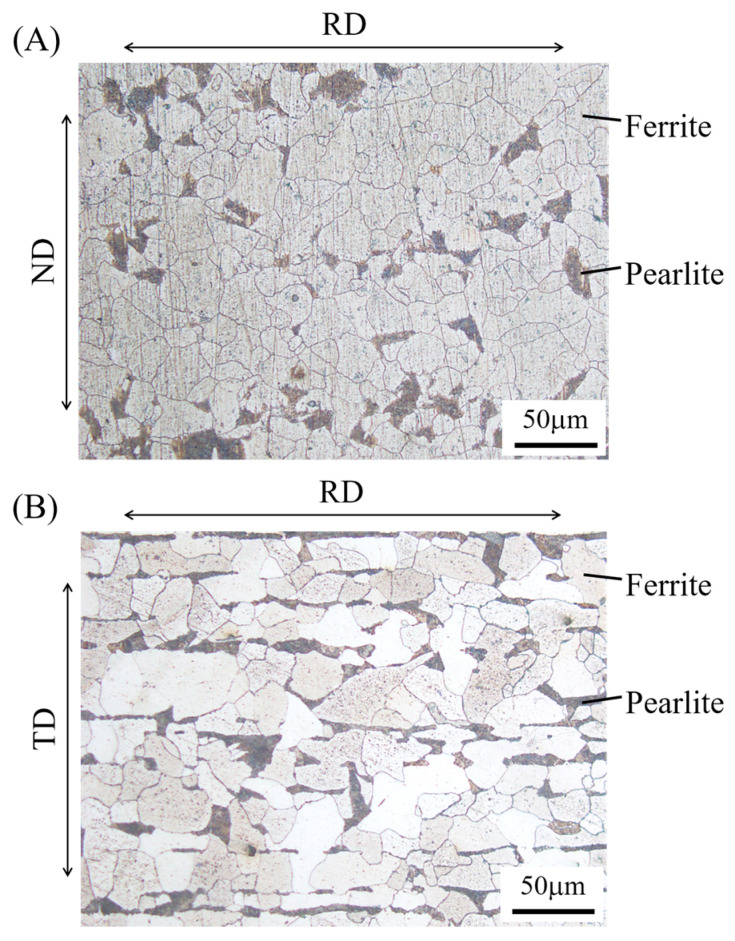
Microstructure of the steel used in this study: (**A**) upper surface and (**B**) side surface of an as-received steel plate. Note: TD—thickness direction, ND—normal direction, and RD—rolling direction.

**Figure 3:**
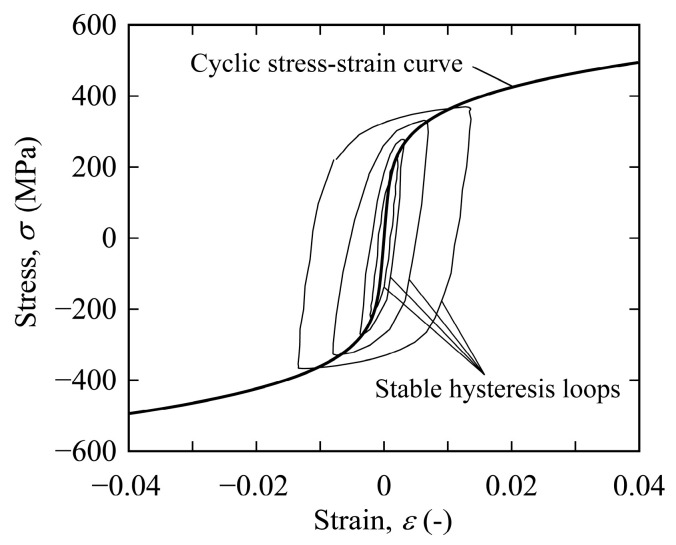
Cyclic stress−strain curve of the steel.

The material used in this study was hot-rolled SPV235 (Nippon Steel Corp., Tokyo, Japan), which is a steel for pressure vessels for intermediate-temperature service in accordance with the Japanese Industrial Standard (JIS) G3115 [20]. Figure 2 shows the microstructure of the steel, which was etched with 3% Nital. The steel consists of ferrite and pearlite, and these grains are elongated along the rolling direction, which forms a so-called ferrite band. Table 1 and Table 2 show the chemical composition and mechanical properties, respectively, of the steel used. Figure 3 shows the cyclic stress–strain curve of the steel.

**Figure 4:**
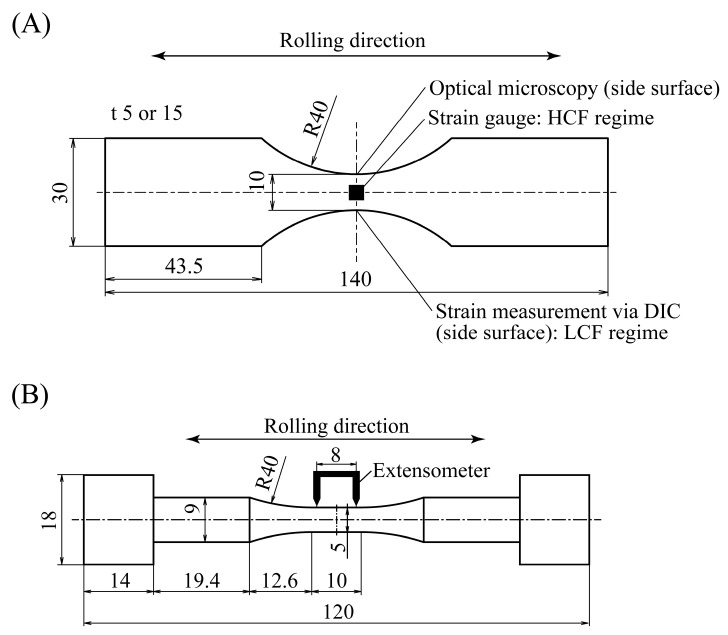
Dimensions of specimens (in units of mm): (**A**) bending specimen and (**B**) tension–compression (T/C) specimen.

In this study, we conducted cyclic alternating bending tests and cyclic T/C tests to investigate the influence of the strain gradient on the fatigue life. Plane bending specimens with thicknesses of *t* = 5 and 15 mm were used for the bending tests, while a cylindrical bar specimen with a parallel section was used for the T/C tests. The dimensions of these specimens are shown in Figure 4. Their longitudinal direction was set to be the rolling direction of the steel. The specimens were machined from as-received steel plates with a thickness of 22 mm, and all surfaces of the specimens were ground with sandpaper having grits up to #800 before testing.

### 2.2. Alternating Bending Testing

**Figure 5:**
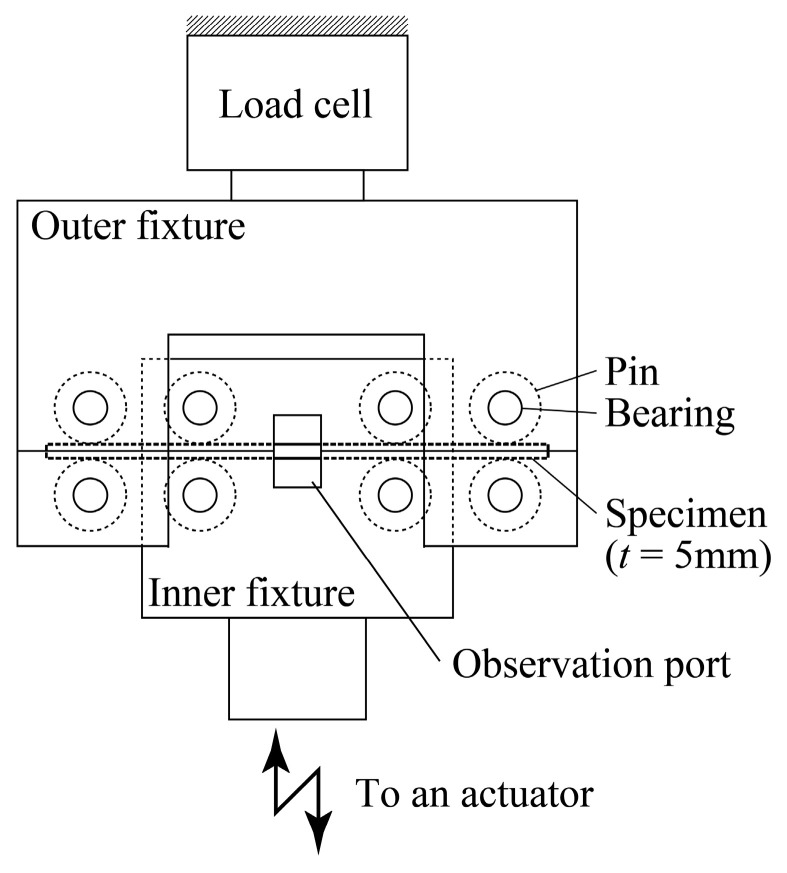
Schematic illustration of a four-point bending jig for a specimen with a thickness of *t* = 5 mm.

The cyclic bending tests developed by the authors were conducted. Figure 5 shows a schematic illustration of a testing apparatus for a specimen with a thickness of *t* = 5 mm. The specimen is mounted between cylindrical pins with a diameter of 20 mm, which can freely rotate via roller bearings attached to upper and lower fixtures. The inner and outer spans are 60 and 120 mm, respectively.

The inner fixture is attached to an actuator that cyclically moves upward and downward, and the specimen is subjected to cyclic alternating bending. As for the specimen with a thickness of *t* = 15 mm, fatigue tests were conducted by changing the diameter of the pins in Figure 5. In a plastically deformed specimen subjected to severe bending, stress, elastic strain, and plastic strain cannot be experimentally determined; therefore, we proposed a technique to determine these values by combining an experiment and FEA, as explained in the introduction. Note that the maximum stress and strain occur at the edge of the specimen subjected to bending, and the stress and strain at the specimen edge are used to evaluate fatigue life because it is reasonable to characterize fatigue life using the stress/strain at a site where the maximum stress/strain occurs. These values were derived based on the technique that is briefly explained in Appendix A.

When the maximum stress at the specimen edge was below approximately 350 MPa, fatigue tests were conducted with a load ratio of −1 and frequency of 10 Hz. The testing was terminated when the maximum displacement exceeded ±2 mm. In contrast, when the maximum stress at a specimen edge was above approximately 350 MPa, fatigue tests were conducted with a displacement ratio of −1 and frequency of 1, 0.5, or 0.1 Hz. The testing was terminated when the load decreased by more than 50% from the maximum load during testing. Note that the tested specimens were found to be almost fractured under these termination conditions. The fatigue tests were carried out in air at room temperature with an electro-hydraulic fatigue testing apparatus (EHF-EM50kN-10L, Shimadzu Corp., Kyoto, Japan).

The relationship between load and strain at the specimen edge was obtained during testing. As for the HCF testing, the strain was measured with strain gauges attached to the upper and lower surfaces of the specimen, as shown in Figure 4A. The strain at the specimen edge was not measured and instead was estimated from the strain at the center of the upper and lower surfaces, taking into account the strain concentration calculated by FEA, which is also explained in Appendix A. For the LCF testing, the strain at the specimen edge was experimentally measured via the digital image correlation (DIC) technique [21]. Prior to fatigue testing, a random pattern consisting of micron-sized particles was painted onto the side surface using black and white spray paint. During testing, images of the side surface were periodically recorded by a digital camera (D7200, Nikon, Tokyo, Japan). The open-source DIC software Ncorr [22] was used for strain calculation. It should be noted that these procedures of the strain measurement by strain gauge and DIC technique are reasonable and their validity has previously been evaluated [18].

### 2.3. Tension–Compression Testing

Cyclic T/C tests were conducted under displacement-controlled conditions with a displacement ratio of −1 and frequency of 0.1 Hz in the LCF and HCF regimes. An extensometer with a gauge length of 8 mm was attached to a parallel portion of the specimen, as shown in Figure 4B, and the displacement was measured during testing. The ranges of stress, elastic strain, and plastic strain were determined based on uniaxial stress–strain hysteresis curves. Note that the stress and strain of the T/C specimen can be experimentally derived as the applied load divided by the original section at the parallel portion and the displacement divided by the gauge length, respectively, which is different from the case of the bending specimen. The testing was also terminated when the load dropped by more than 50% from the maximum load.

### 2.4. Fatigue Life Evaluation Based on Strain-Based Approach

Some of these tests were periodically interrupted, and the specimen surface was observed with replica technique and optical microscopy to investigate crack initiation and propagation. In the bending specimens, the side surfaces were observed, and the crack depth, *b*, was measured as the distance from the top/bottom surface to the crack tip. On the other hand, in the T/C specimens, the surface was observed, and the crack length, 2*a*, was measured as the projected length on the plane perpendicular to the loading direction. The observation interval was set as the number of roughly 1/20 of the estimated total failure life, *N_f_*, of the specimen, irrespective of loading condition.

The crack initiation life, *N_i_*, and total failure life, *N_f_*, were characterized by the strain-based approach: the relationships between elastic strain range and life (Δ*ε_e_*–*N*) and between plastic strain range and life (Δ*ε_p_*–*N*) were characterized by the Basquin law, as in Equation (1), and the Coffin–Manson law, as in Equation (2), respectively:

(1) $$\Delta \varepsilon_{e}=C_{e}N^{-\alpha },$$

(2) $$\Delta \varepsilon_{p}=C_{p}N^{-\beta },$$

where *C_e_*, *C_p_*, *α*, and *β* are constants. According to Equations (1) and (2), the relationship between total strain range and life (Δ*ε_t_*–*N*) is given by the following:

(3) $$\Delta \varepsilon_{t}=\Delta \varepsilon_{e}+\Delta \varepsilon_{p}=C_{e}N^{-\alpha }+C_{p}N^{-\beta }.$$

Note that although some advanced equations such as Morrow [23], Smith–Watson–Topper [24], and Manson–Halford models [25] have been proposed to predict the fatigue life of metals, they were not used in this study. This is because the purpose of this study is to evaluate the influence of the strain gradient on the fatigue life, not to investigate an equation that can predict the life with high accuracy.

## 3. Experimental Results and Discussion

### 3.1. Evaluation of Crack Initiation and Total Failure Lives

**Figure 6:**
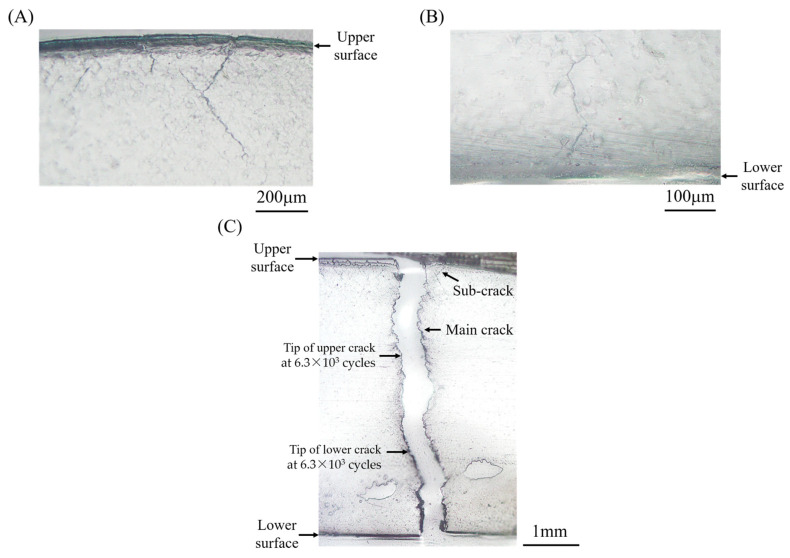
Crack profiles (bending specimen, *t* = 5 mm, Δ*ε_t_* = 0.015, and *N_f_* = 6.5 × 10^3^ cycles): (**A**) crack propagation from upper surface, *N/N_f_* = 0.87, (**B**) crack propagation from lower surface, *N/N_f_* = 0.87, and (**C**) final fracture due to coalescence of cracks propagating from upper and lower surfaces.

Figure 6 shows some edge cracks that occurred in the bending specimen (*t* = 5 mm) subjected to Δ*ε_t_* = 0.015, which was obtained using the replica technique. At the number of cycles *N*/*N_f_* = 0.87, cracks were observed at the upper and lower surfaces and propagated from the surfaces toward the specimen center (see Figure 6A,B). Note that some cracks were initiated, which is clearly observed at the upper surface shown in Figure 6A. Then, cracks propagating from the upper and lower surfaces coalesced around the center, and the specimen was fractured (see Figure 6C). The sites of crack tips just before fracture (*N* = 6.3 × 10^3^ cycles) are indicated in this figure. The upper and lower cracks were almost equal in length, and it was considered that the cracks coalesced in the center of the specimen, and the specimen was fractured. These trends in crack initiation, propagation, and fracture were the same as those in the other specimens, irrespective of specimen thickness.

**Figure 7:**
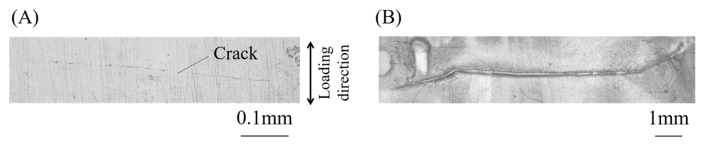
Crack profiles obtained using the replica technique (T/C specimen, Δ*ε_t_* = 0.03, and *N_f_* = 5.0 × 10^2^ cycles): (**A**) crack that was initially observed at *N/N_f_* = 0.12 and (**B**) crack at final fracture (*N/N_f_* = 1).

Figure 7 shows a crack occurring in the T/C specimen subjected to Δ*ε_t_* = 0.028. A crack along the plane perpendicular to the loading direction was initially observed at *N/N_f_* = 0.12 (see Figure 7A). Then, the crack propagated along the plane, and the specimen was fractured, as shown in Figure 7B. These trends were almost the same for all T/C specimens within the scope of this study. Comparing these results, the fatigue processes are summarized as follows. In the bending specimens, cracks occurred at the upper and lower surfaces and propagated, and the fracture was caused by the coalescence of these cracks around the specimen center. On the other hand, in the T/C specimens, a crack occurred and propagated along the plane perpendicular to the loading direction, and the fracture occurred.

**Figure 8:**
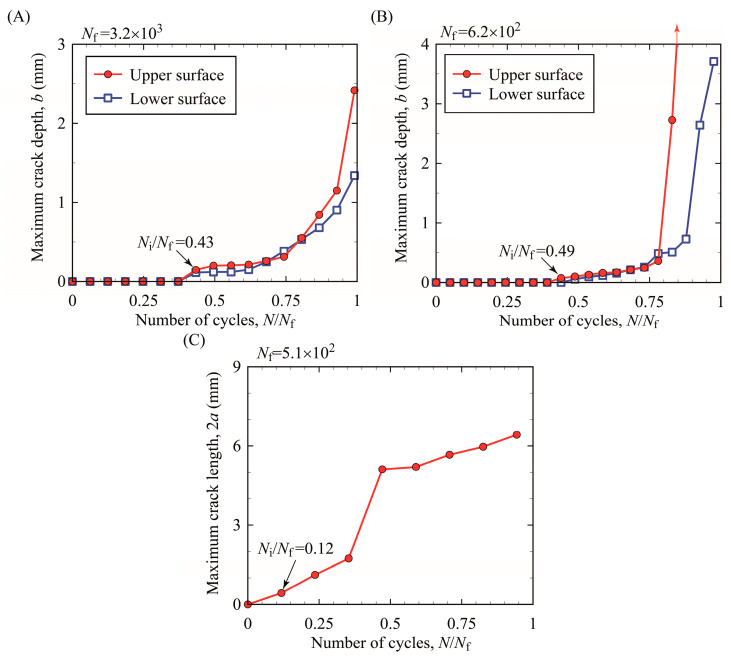
Crack initiation and propagation behavior: (**A**) bending testing (*t* = 5 mm) at Δ*ε_t_* = 0.02, (**B**) bending testing (*t* = 15 mm) at Δ*ε_t_* = 0.028, and (**C**) T/C testing at Δ*ε_t_* = 0.028.

The length of cracks was measured to compare crack initiation and propagation lives of bending and T/C specimens. Figure 8 shows the changes in crack lengths in the specimens subjected to a total strain range, Δ*ε_t_*, of approximately 0.02. In the horizontal axis, the number of cycles, *N*, is normalized by the number of cycles to total failure, *N_f_*. As multiple cracks occurred, the depth/length of the deepest/longest crack was measured for each specimen. The number of cycles to crack initiation is also shown in this figure, defined as the number of cycles where a crack longer than 0.1 mm was first observed. The crack length/depth of 0.1 mm corresponds to a micro-crack propagating only through a few grains, which would be reasonable for the crack initiation condition from a microstructural viewpoint [26]. In the specimens subjected to cyclic bending, cracks occurred at both surfaces at nearly a half of total failure life (*N*/*N_f_* ≃ 0.5), and then, cracks at the upper and lower surfaces propagated rapidly after *N*/*N_f_* ≃ 0.75. On the other hand, in the T/C specimen, a crack occurred very early, propagated steadily, and then, the total failure occurred. Note that the crack initiation life of the T/C specimen shown in Figure 8C was estimated as *N*/*N_f_* = 0.12, which might be an overestimation because the observation interval is not sufficient as in other specimens.

**Figure 9:**
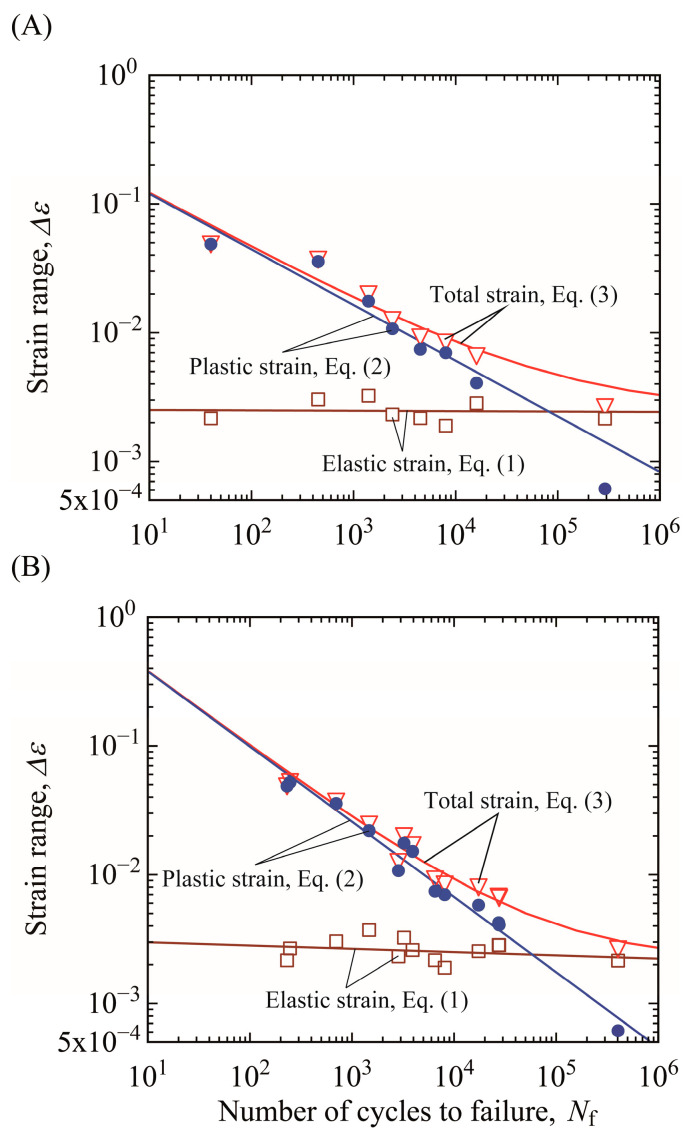
Strain-based approach for bending testing (*t* = 5 mm): (**A**) crack initiation life, and (**B**) total failure life.

Figure 9A,B shows the relationship between strain range at the specimen edge and crack initiation and total failure lives (Δ*ε*–*N_i_* and Δ*ε*–*N_f_* relations), respectively. The Δ*ε_e_*–*N_i_* and Δ*ε_e_*–*N_f_* relations and the Δ*ε_p_*–*N_i_* and Δ*ε_p_*–*N_f_* relations were fitted based on the least-squares technique using Equations (1) and (2), respectively. It should be noted that the total strain range Δ*ε_t_* is defined as the sum of elastic strain range, Δ*ε_e_*, and plastic strain range, Δ*ε_p_*, so that it is not necessary to fit the Δ*ε_t_*–*N* relation directly using Equation (3). The Δ*ε_e_*–*N_i_* and Δ*ε_e_*–*N_f_* relations can be described by straight lines and agree with the Basquin law, and the Δ*ε_p_*–*N_i_* and Δ*ε_p_*–*N_f_* relations can be also described by straight lines and agree with the Coffin–Manson law. These trends were also confirmed for the other loading conditions. This implies that the strain-based approach is suitable for evaluating the crack initiation and total failure lives of mechanical components using the strain range at the site where maximum strain occurs even if the strain gradient occurs in the components. That is, it is reasonable to evaluate the influence of the strain gradient on the fatigue lives using the strain-based approach.

### 3.2. Influence of Strain Gradient on Fatigue Life

**Figure 10:**
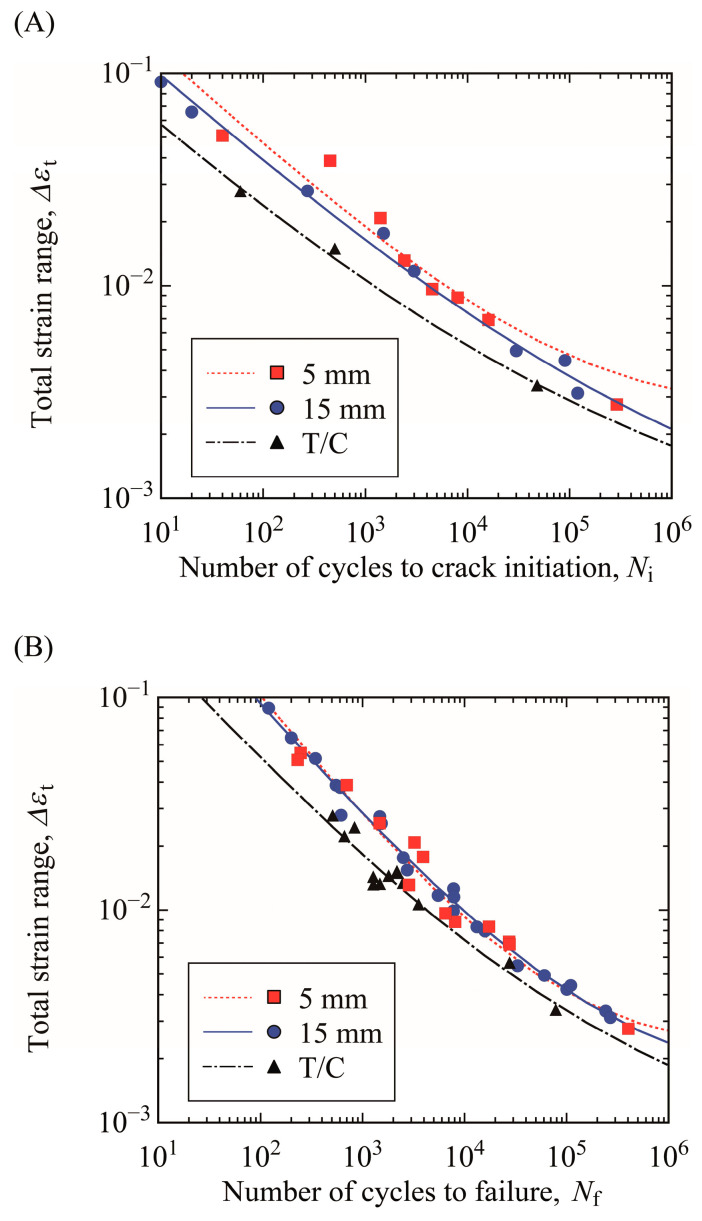
Δ*ε_t_*-*N* relations of bending and T/C testing: (**A**) crack initiation life *N_i_* and (**B**) total failure life *N_f_*.

Figure 10A,B shows the relationships between total strain range and crack initiation life and between total strain range and total failure life (Δ*ε_t_*–*N_i_* and Δ*ε_t_*–*N_f_* relations), respectively. The curve representative of fatigue life for each loading condition is drawn using the strain-based approach of Equation (3) derived from Equations (1) and (2). The crack initiation life of T/C testing was the shortest in the test conditions in this study. The crack initiation life of the specimen with a thickness of 15 mm was slightly shorter than that of the specimen with a thickness of 5 mm from the LCF to HCF regimes, indicating that the influence of plate thickness on crack initiation life must be considered. The total failure life of T/C testing was also the shortest. However, the total failure life of the specimen with a thickness of 15 mm was almost the same as that of the specimen with a thickness of 5 mm.

The crack initiation life was shorter in the thicker bending specimen although the total failure life of the bending specimens with different thicknesses was almost the same. Note that the total failure life is the sum of crack initiation life and crack propagation life. Hence the crack propagation life was longer in the thicker bending specimen. This can be explained from a viewpoint of crack propagation behavior. Fatigue cracks occurred at both the upper and lower surfaces of each bending specimen and propagated toward the specimen center. Then, these cracks coalesced at the center, indicating that the fatigue fracture occurred, as shown in Figure 6C. Hence, the crack depth, *b*, at fracture was approximately half of the specimen thickness, *t*. From a mechanical viewpoint, this situation is similar to a typical single-edge-cracked bending specimen because it is considered that cracks on the tensile side are opened whereas cracks on the compressed side are closed during testing. Generally, the fracture mechanics parameter governing crack propagation is influenced by bending stress/strain, crack depth *b*, and ligament *t* − *b* in the single-edge-cracked specimen [27,28]. If the ligament is wide enough, the fracture mechanics parameter is little influenced by the ligament. This means that the fracture mechanics parameter for specimens subjected to bending with different thicknesses, same crack length, and wide ligament is almost the same if the specimen is subjected to the same bending stress/strain. Consequently, the propagation rate of the cracks with the same length would be almost the same, irrespective of specimen thickness. The results obtained in this study can be understood as follows: from crack initiation at a smooth surface to total failure, a crack propagated by 2.5 mm (=0.5*t*) for the thinner specimen (*t* = 5 mm) whereas a crack propagated by 7.5 mm (=0.5*t*) for the thicker specimen (*t* = 15 mm). From the foregoing discussion, for these cracks the number of cycles required for the crack propagation up to 2.5 mm was considered to be almost the same, irrespective of specimen thickness. At this point, the thinner specimen (*t* = 5 mm) was fractured, although the thicker specimen (*t* = 15 mm) was not fractured (the cracks must propagate by the remaining 5 mm for fracture). The crack propagation life was longer because the distance of crack propagation required for final fracture was longer in the thicker specimen subjected to cyclic bending. As a result, it might be a coincidence that the total failure lives of thick and thin specimens, which are the sum of crack initiation and propagation lives, were similar.

Figure 10A revealed that the specimen thickness affected the crack initiation life in the LCF and HCF regimes. To evaluate this size effect, the strain gradient, *χ*, at the tensile edge of the specimen subjected to maximum bending is defined by the following equation:

(4) $$\chi =\frac{1}{\varepsilon_{t}}{\frac{d\varepsilon_{t}}{dt}|}_{\mathrm{edge}}.$$

**Figure 11:**
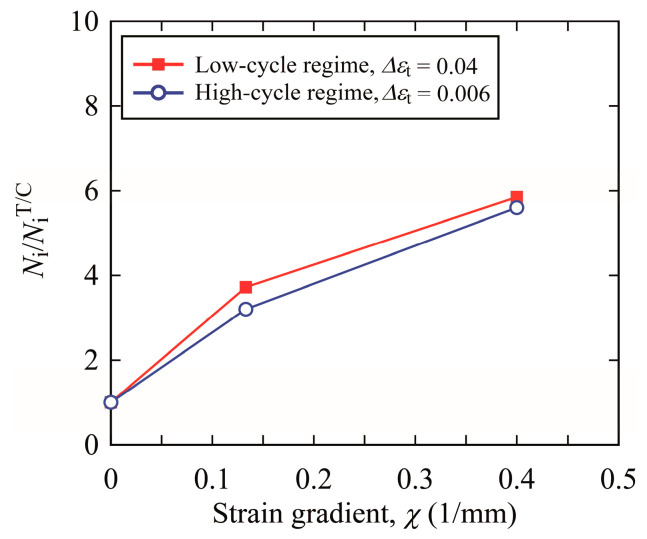
Influence of strain gradient on crack initiation life.

In the case of the T/C specimen, *χ* became null due to no strain gradient in the cross-section of the specimen. Figure 11 shows the relationship between crack initiation life, *N_i_*, and strain gradient, *χ*, under the conditions of Δ*ε_t_* = 0.04 (LCF regime) and 0.006 (HCF regime). In the vertical axis, the crack initiation life, *N_i_*, is normalized by that for the T/C testing, *N_i_*^T/C^. The crack initiation life, *N_i_*/*N_i_*^T/C^, increased with increasing strain gradient, and the increasing trend seems to be almost the same. Hence, it was found that the crack initiation life must be evaluated using strain and its gradient in LCF and HCF regimes.

When the bending stress is below the yield stress of metals, the stress is proportional to the strain. Hence, the influence of strain gradient on crack initiation life would be the same as that of stress gradient, which is a so-called size effect, as mentioned in the introduction. On the other hand, if the bending stress is above the yield stress, the stress gradient becomes gentle near the surfaces due to plastic deformation. If the stress gradient affected the fatigue life, the fatigue life of the bending specimen should become closer to that of the T/C specimen, but this was not the case. Hence, life evaluation based on strain and its gradient, not stress and its gradient, is effective in LCF and HCF regimes.

In general, the fatigue life of a component in machines and structures is evaluated by fatigue tests using smooth specimens subjected to cyclic T/C, as mentioned in the introduction. In these tests, the strain gradient in a component is ignored, and the fatigue life may be incorrectly underestimated (that is, the fatigue life is estimated to be shorter than it actually is). As shown in Figure 11, when the strain gradient becomes steep, the crack initiation is delayed, indicating that the crack initiation life is prolonged. On the other hand, as for the crack propagation life, the life can be evaluated accurately by the fracture mechanics approach. We can obtain the fatigue crack propagation characteristics of many structural metals in the handbook [29]. To evaluate the service life of a component accurately, it is necessary to evaluate the fatigue crack initiation life considering not only the strain but also its gradient at the site where the maximum strain occurs, which would be the crack initiation site. The validity of this approach to evaluate crack initiation life using strain and its gradient was investigated only within the scope of this study and needs to be evaluated for various metals. In addition, since the fatigue behavior is very scattered, it is necessary to carry out many experiments and to statistically evaluate the fatigue life [30,31]. It is also necessary to formulate a life evaluation equation based on the Basquin and Coffin–Manson equations. These will be the next steps in this research.

## 4. Conclusions

This study investigated the influence of the strain gradient on the fatigue life of the steel used for pressure vessels. Alternating bending fatigue tests were carried out using specimens with different thicknesses, and cyclic T/C tests were also done. During testing, crack initiation and propagation behavior were observed, and the lives of crack initiation and propagation were evaluated with a strain-based approach. The following conclusions were obtained:

1. The strain-based approach based on the forms of the Basquin and Coffin–Manson equations was effective for evaluating fatigue life, irrespective of loading condition.
2. The crack initiation life was affected by not only strain but also the strain gradient at the crack initiation site, and the crack initiation life increased with increasing strain gradient.
3. The total failure life of the specimen subjected to cyclic T/C was the shortest, while the total failure life of the specimens with thicknesses of 5 and 15 mm subjected to alternating bending was almost the same. This is because the crack initiation life and crack propagation life become shorter and longer, respectively, with increasing thickness of the bending specimen.

## Figures and Tables

**Table 1:** Chemical composition of the steel (mass%), which was provided by the manufacturer.

C	Si	Mn	P	S	Cu	Ni	Cr
0.12	0.22	1.04	0.015	0.004	0.01	0.02	0.03

**Table 2:** Mechanical properties of the steel.

Young’s Modulus, GPa	Poisson’s Ratio	Yield Stress, MPa	Tensile Strength, MPa
215	0.32	247	430

**Figure A1:**
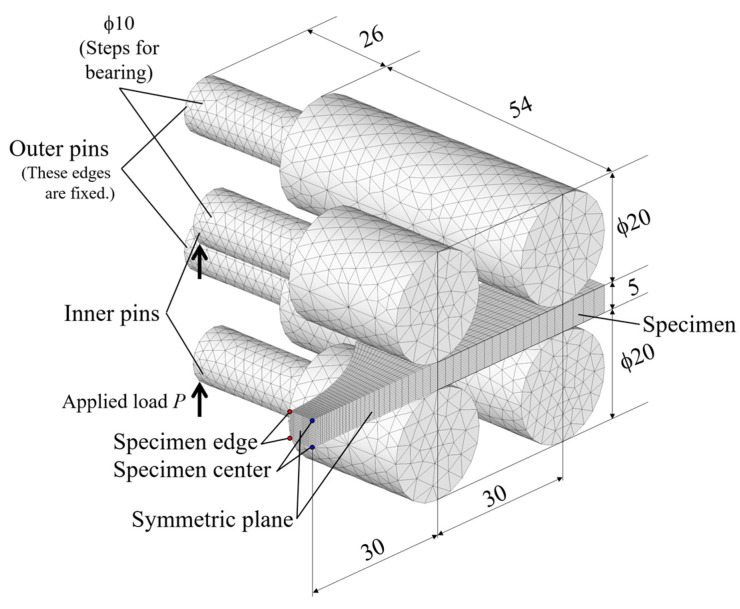
Finite element model and boundary conditions of a four-point bending specimen with thicknesses of 5 mm.

**Figure A2:**
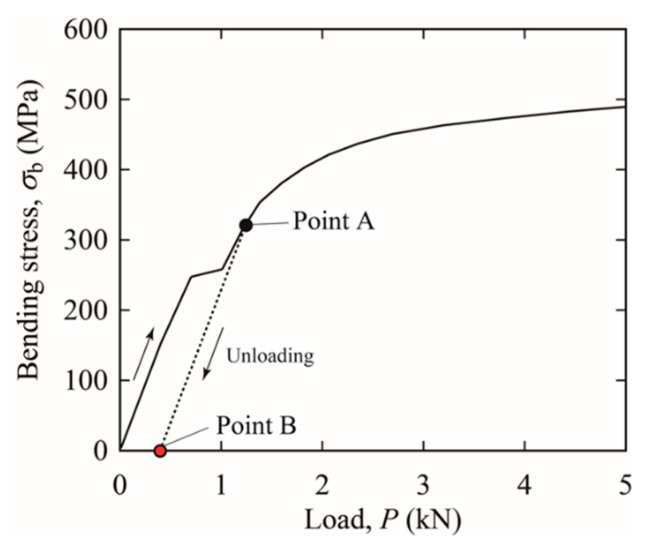
Relationship between load, *P*, and bending stress, *σ_b_*, at the specimen edge obtained based on FEA.

**Figure A3:**
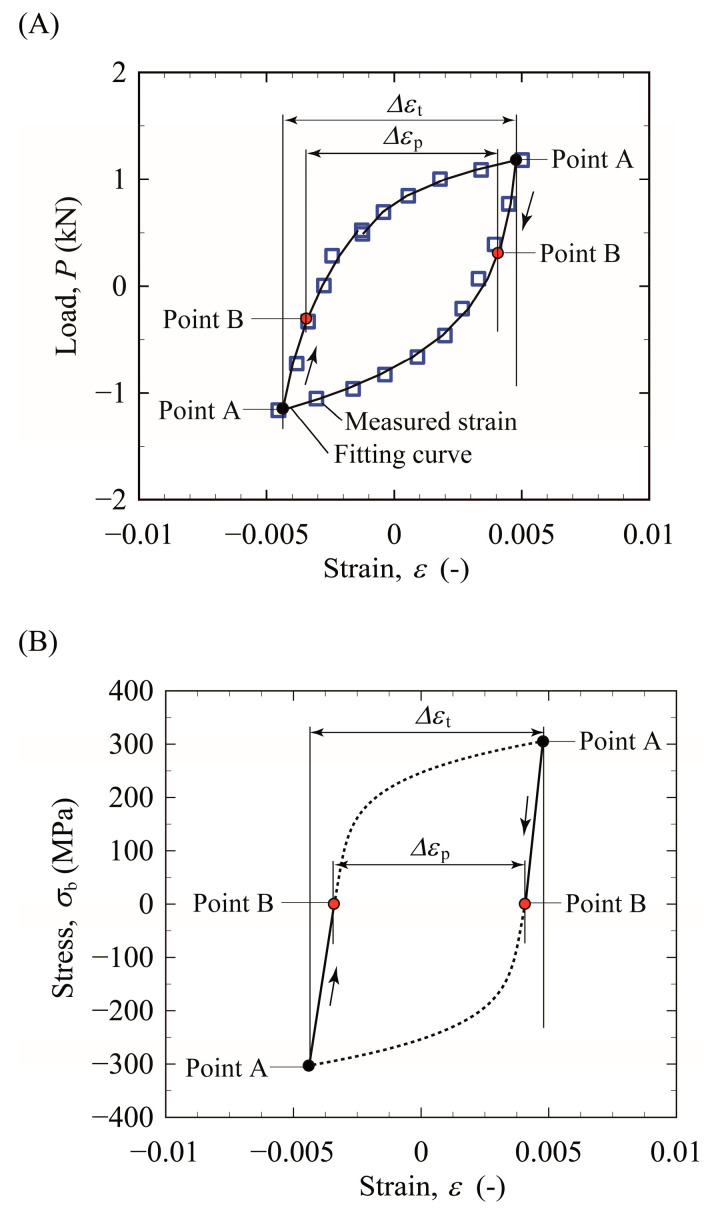
Technique to estimate the stress–strain curve at the specimen edge from the load–strain curve obtained by experiment under Δ*ε_t_* = 0.01 (*N/N_f_* = 0.77): (**A**) load–strain curve obtained experimentally and (**B**) estimated stress–strain curve.

**Figure A4:**
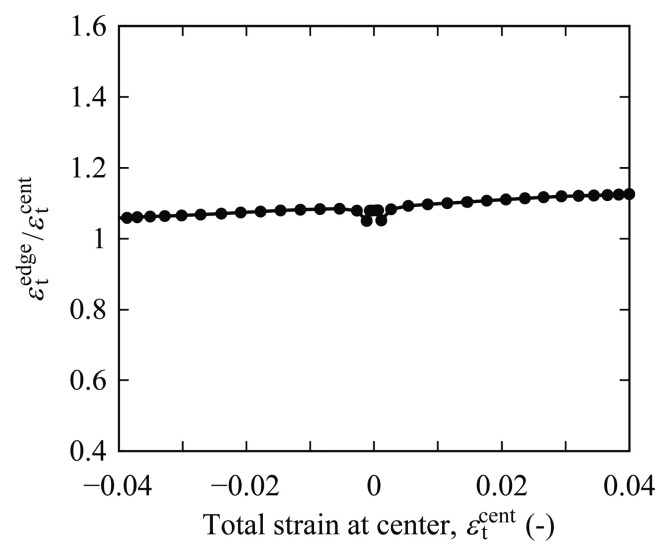
Strain concentration of a specimen with 5 mm thickness subjected to bending. The values of *ε_t_*^edge^ and *ε_t_*^cent^ represent the strains at the specimen edge and the specimen center, respectively, shown in Figure A1.

## Data Availability

The data presented in this study are available on request from the corresponding author.

## Appendix A

A previously developed four-point bending technique was applied to the investigation of alternating bending fatigue properties. In this appendix, the specimen with a thickness of 5 mm is briefly explained. For details, see the previous study [18]. In this technique, stress, elastic strain, and plastic strain during testing are estimated based on the relationship between load and total strain, which is experimentally measured, and the relationship between load and stress, which is obtained using elastic–plastic FEA.

Figure A1 shows a quarter model of the four-point bending specimen (*t* = 5 mm) for elastic–plastic FEA using ANSYS 19.0. For simulating actual test jigs, the outer pins were fixed, and the inner pins were moved upward and downward in turn. The pins and specimen were treated as elastic and elastic–plastic bodies, respectively. The elastic–plastic analysis was conducted using the cyclic stress–strain curve shown in Figure 3. In the contact analysis, the friction coefficient was set at null because the roller bearings could freely rotate.

Figure A2 shows the relationship between load, *P*, and stress, *σ_b_*, at the specimen obtained via FEA. If elastic unloading occurs at point A, the stress shifts from point A to point B. At point B, the load is not null, whereas the stress is null. Based on a hysteresis loop between load, *P*, and strain, *ε_t_*, measured experimentally, a stress–strain hysteresis loop was estimated. Figure A3A shows the *P* − *ε_t_* hysteresis loop of the specimen, which was obtained from the test conducted under Δ*ε_t_* = 0.01 at *N*/*N_f_* = 0.77. The strains were measured via DIC, and fitting curves based on these measured data were drawn using exponential functions. The stress amplitude, Δ*σ_b_*/2, could be estimated as the stress at point A in Figure A2. Point B, corresponding to load for zero stress, could be plotted on the unloading curve from point A. As a result, the points A and B were plotted on the *P* − *ε_t_* curve shown in Figure A3A. This same procedure was applied to the opposite loading direction, and the points A and B were also plotted in the reverse side, as shown in Figure A3A. The plastic strain range, Δ*ε_p_*, was obtained as a range between points B and the elastic strain range, Δ*ε_e_*, is calculated using the following relation:

(A1) $$\Delta \varepsilon_{e}=\Delta \varepsilon_{t}-\Delta \varepsilon_{p}.$$

According to this procedure, the stress–strain hysteresis at the specimen edge could be drawn, as shown in Figure A3B. Note that this procedure can derive only the unloading curves from the maximum stress to zero stress. The curves from zero to the maximum stress were estimated as smooth curves from zero to the maximum stress (dotted lines) by considering the cyclic stress–strain curve. This was not a problem because the curves were not used for the life evaluation via the strain-based approach.

As shown in Figure 4A and Figure A1, the strain was measured at the specimen center in the HCF regime with a strain gauge, and the strain was measured at the specimen edge in the LCF regime by DIC. Due to the constricted shape of the specimen, the strain at the specimen edge was a little different from that at the specimen center. Hence, the stress/strain distributions at the upper and lower edges were evaluated. Figure A4 shows the relationship between total strain at the specimen edge and that at the specimen center obtained by FEA. Using this relationship, the strain at the specimen edge was estimated based on the strain measured with a strain gauge to characterize the fatigue life using a strain-based approach.
